# Supplementary material for: Syndemic interactions between HIV/AIDS, mental health conditions, and non-communicable diseases in sub-Saharan Africa: A scoping review of contributing factors
Source: PLoS One. 2025 Aug 21;20(8):e0328515. doi: 10.1371/journal.pone.0328515 (PMC12370135; doi:10.1371/journal.pone.0328515)
Supplement: S1 Appendix — For our search strategy, the search items included: Non-communicable Diseases (i.e., Cardiovascular Disease, Cancer, Kidney Disease, Respiratory Diseases, and Diabetes), Mental Health (i.e., Mood disorders, substance-use disorders, anxiety disorders, Stigma), and People living with HIV. The search limits included the following: publication year of 2000–2024, no language restrictions, geography of Sub-saharan Africa, and studies focusing specifically on examining the impact of HIV and mental health on the prevalence and management of Non-communicable Diseases. (DOCX) [file pone.0328515.s001.docx]

S1 Appendix. Search Strategy.

**Title of Review:** Syndemic Interactions between HIV/AIDS, Mental Health Conditions, and Non-Communicable Diseases in sub-Saharan Africa: A Scoping Review of Contributing Factors

**Countries of Interest:** Angola, Benin, Botswana, Burkina Faso, Burundi, Cabo Verde, Cameroon, Central African Republic, Chad, Comoros, Republic of the Congo, Democratic Republic of the Congo, Côte d'Ivoire, Djibouti, Equatorial Guinea, Eritrea, Eswatini, Ethiopia, Gabon, The Gambia, Ghana, Guinea, Guinea-Bissau, Kenya, Lesotho, Liberia, Madagascar, Malawi, Mali, Mauritania, Mauritius, Mozambique, Namibia, Niger, Nigeria, Rwanda, São Tomé and Príncipe, Senegal, Seychelles, Sierra Leone, Somalia, South Africa, South Sudan, Sudan, Tanzania, Togo, Uganda, Zambia, and Zimbabwe

**Database:** PubMed/MEDLINE

| **Search Strategy** |
| --- |
| (HIV Infections OR HIV infections OR AIDS OR acquired immunodeficiency syndrome) AND (Mental health OR mental illness OR mental disorders OR anxiety OR depression OR stress OR affective disorders OR problem behavior OR mental fatigue OR psychological stress OR burnout OR occupational stress OR paranoid behavior OR neurodevelopmental disorders OR Schizophrenia OR psychotic disorder OR psychotic disorders OR catatonia OR mood disorders OR fear-related disorders OR obsessive-compulsive disorders OR OCD OR dissociative disorder OR feeding disorder OR eating disorder OR elimination disorder OR substance-use OR addictive behavior OR dementia OR delirium OR psychosis OR factitious disorder OR impulsive-control disorder OR mania OR post-traumatic stress disorder OR personality disorder OR anxiety OR depression OR neurocognitive OR neurocognitive disorder OR neurotic disorder OR cognitive OR psychiatric OR psychiatric disorders OR psychiatric disorder OR psychiatry OR psychology OR psychosocial OR alcohol dependence OR alcohol abuse OR substance abuse OR substance-related disorders OR substance-related disorders OR substance dependence OR common mental health disorders OR severe mental health disorders) AND (syndemics OR syndemic OR synergy OR synergistic OR comorbidity OR comorbidities OR multimorbidity OR co-existing disease* [tw]) AND (cardiovascular disease OR heart attacks OR myocardial infarction OR stroke OR coronary heart disease OR cardiometabolic disease OR tuberculosis OR TB OR diabetes OR metabolic syndrome OR chronic disease OR cancer OR chronic kidney disease OR end-stage renal disease OR asthma OR ischemic heart disease OR rheumatic heart disease OR congenital heart disease OR peripheral arterial disease OR deep vein thrombosis OR pulmonary embolism OR cerebrovascular disease OR disease clusters OR chronic diseases OR noncommunicable diseases OR noncommunicable disease OR non-infectious diseases OR non-infectious disease OR non-communicable chronic disease OR non-communicable chronic diseases) AND ("Africa South of the Sahara"[Mesh] OR Africa [tw] OR African[tw]) |

**Database:** Global Health, PsycINFO (ProQuest), PsycINFO (OVID)

| **Search Strategy** |
| --- |
| (HIV Infections OR HIV infection OR AIDS OR acquired immunodeficiency syndrome OR HIV coinfection OR HIV OR human immunodeficiency virus) AND (Mental health OR mental illness OR mental disorders OR anxiety OR depression OR stress OR affective disorders OR problem behavior OR mental fatigue OR psychological stress OR burnout OR occupational stress OR paranoid behavior OR neurodevelopmental disorders OR Schizophrenia OR psychotic disorder OR psychotic disorders OR catatonia OR mood disorders OR fear-related disorders OR obsessive-compulsive disorders OR OCD OR dissociative disorder OR feeding disorder OR eating disorder OR elimination disorder OR substance-use OR addictive behavior OR dementia OR delirium OR psychosis OR factitious disorder OR impulsive-control disorder OR mania OR post-traumatic stress disorder OR personality disorder OR anxiety OR depression OR neurocognitive OR neurocognitive disorder OR neurotic disorder OR cognitive OR psychiatric OR psychiatric disorders OR psychiatric disorder OR psychiatry OR psychology OR psychosocial OR alcohol dependence OR alcohol abuse OR substance abuse OR substance-related disorders OR substance-related disorders OR substance dependence OR common mental health disorders OR severe mental health disorders) AND (syndemics OR syndemic OR synergy OR synergistic OR comorbidity OR comorbidities OR multimorbidity OR multimorbidities OR co-existing disease) AND (cardiovascular disease OR heart attacks OR myocardial infarction OR stroke OR coronary heart disease OR cardiometabolic disease OR diabetes OR metabolic syndrome OR chronic disease OR cancer OR chronic kidney disease OR end-stage renal disease OR asthma OR ischemic heart disease OR rheumatic heart disease OR congential heart disease OR peripheral arterial disease OR deep vein thrombosis OR pulmonary embolism OR cerebrovascular disease OR disease clusters OR chronic diseases OR noncommunicable diseases OR noncommunicable disease OR non-infectious diseases OR non-infectious disease OR non-communicable chronic disease OR non-communicable chronic diseases) AND (Africa OR African OR Algeria OR Algerian OR Egypt OR Egyptian OR Libya OR Libyan OR Morocco OR Moroccan OR Tunisia OR Tunisian OR Angola OR Angolan OR Benin OR Botswana OR Burkina Faso OR Burkinabe OR Burundi OR Burundian OR Cabo Verde OR Central African Republic OR Central Africa OR Central African OR Cameroon OR Chad OR Chadian OR Congo OR Congolese OR Comoros OR Cote d’Ivoire OR Ivory Coast OR Djibouti OR Equatoria Guinea OR Eswatini OR Gabon OR Ghana OR Ghanaian OR Sao Tome OR Principe OR Eritrea OR Eritrean OR Ethiopia OR Ethiopian OR Gambia OR Gambian OR guinea OR Guinean OR guinea Bissau OR Bissau Guinean OR Kenya OR Kenyan OR Lesotho OR Liberia OR Liberian OR Madagascar OR Malagasy OR Malawi OR Malawian OR Mali OR Malian OR Mozambique OR Mozambican OR Mauritania OR Mauritanian OR Namibia OR Namibian OR Niger OR Nigerian OR Northern Africa OR Rwanda OR Rwandan OR Senegal OR Senegalese OR Seychelles OR Sierra Leone OR Sierra Leonean OR Somalia OR Somali OR South Africa OR South African OR South Sudan OR Sudan OR Sudanese OR Tanzania OR Tanzanian OR Togo OR Togolese OR Uganda OR Ugandan OR Zambia OR Zambian OR Zimbabwe) |

**Database:** Web of Science (all databases), Web of Science (core collection), CINAHL

| **Search Strategy** |
| --- |
| (HIV infections OR AIDS OR acquired immunodeficiency syndrome) AND (Mental health OR mental illness OR mental disorders OR anxiety OR depression OR stress OR affective disorders OR problem behavior OR mental fatigue OR occupational stress OR burnout OR paranoid behavior) AND (Syndemics OR comorbidity OR synergy OR multimorbidity OR co-existing disease OR comorbidities) AND (Cardiovascular disease OR heart attacks OR chronic disease OR cancer OR chronic kidney disease OR non-communicable diseases OR NCDs) AND (Africa OR African OR Sub-saharan Africa) |
