# Supplementary material for: Syndemic interactions between HIV/AIDS, mental health conditions, and non-communicable diseases in sub-Saharan Africa: A scoping review of contributing factors
Source: PLoS One. 2025 Aug 21;20(8):e0328515. doi: 10.1371/journal.pone.0328515 (PMC12370135; doi:10.1371/journal.pone.0328515)
Supplement: Attachment — Submitted filename: Response to Reviewers.docx [file pone.0328515.s004.docx]

**Author Responses to Editorial and Reviewer Comments.**

We would like to express our sincere gratitude to the reviewers and the editorial team for their careful and thorough review of our manuscript. We greatly appreciate the time, effort, and expertise that went into evaluating our work. In the sections below, we provide detailed, point-by-point responses to each of the editorial and reviewer comments. We have carefully considered all feedback and have revised the manuscript accordingly to address the concerns and suggestions raised.

We are especially thankful for the opportunity to revise and resubmit our manuscript. The constructive critiques we received have been instrumental in enhancing the clarity, rigor, and overall quality of the paper. We believe that the manuscript is now substantially improved and hope that the revisions meet your expectations.

**Comments from the Editor**

**Editor Comment 1**

1. Is there any target population for this review-any specific age, gender, etc.? Please clarify. It would be nice to add the names of African Countries to the inclusion criteria.

**Author Response to Comment 1:** Thank you for your thoughtful suggestions. In designing our selection criteria, we intentionally did not impose any specific age or gender restrictions during the selection process, as our aim was to ensure the review was as inclusive and representative of the current literature as possible. By including studies across all age groups and genders, we aimed to ensure that our analysis reflects the diverse and multifaceted nature of syndemic interactions. We specifically sought to examine contributing factors relevant to both male and female individuals to reflect the broader landscape of syndemic interactions.

While all included studies enrolled participants aged 18 and older—with the exception of Carpenter et al.—we did not apply an upper or lower age limit in our inclusion criteria. As noted in **Section 2.1 (Inclusion/Exclusion Criteria),** we have incorporated a note indicating no restrictions were placed on age or gender. Interestingly, the absence of studies involving children and adolescents highlights a clear gap in the current literature. We have now addressed this point explicitly in the **lack of syndemic literature section**, emphasizing the need for future research focused on younger populations, who appear to be underrepresented in syndemics research despite the potential relevance of several non-communicable conditions in this group.

Regarding your second point, given the large number of countries represented in the included studies (approximately 46), we opted to avoid listing them all in the main text to reduce visual clutter. Instead, we have provided the full list of countries in **S1 Appendix SS** for reference.

**Editor Comment 2**

2. In Figure 2, please recheck the calculation—the studies screened (2913) – studies excluded (2706) = 207. The total shown in the figure is 188.

**Author Response to Comment 2:** Thank you for carefully reviewing the figure and bringing this discrepancy to our attention. We have rechecked the calculations and identified the source of the error. The figure has been revised accordingly to accurately reflect the correct number of studies. The updated version is now included in the revised manuscript.

**Editor Comment 3**

3. Scoping reviews do not typically assess the quality of the studies. Usually, systematic reviews appraise studies as one of the core components of their methodology. Figure 6 shows the quality assessment of 13 studies. How about other studies’ findings (for quality)?

**Author Response to Comment 3:** Thank you for the observation. You are correct that formal quality or risk of bias (RoB) assessments are not typically required for scoping reviews, as the primary goal is to map the breadth of evidence rather than evaluate the strength of individual findings. In our case, we conducted a targeted quality assessment of the 13 studies most directly aligned (i.e., qualitative, mixed methods, and cross-sectional) with our objective to provide insight into the methodological rigor of this subset. While we did review all included studies qualitatively for relevance and basic methodological soundness, we did not apply a formal RoB tool to the remaining studies (4), consistent with scoping review methodology. We have clarified this in the manuscript in **section 3.6 Quality assessment** and specified the quality assessment presented in Figure 6.

**Comments from Reviewers**

**Reviewer 1 Comment 1**

1. Line 492-493: Will the figures be expanded in the final print? The legends in the current format are not legible.

**Author Response to Comment 1:** Thank you for this helpful observation. We agree that clarity is essential for effective communication of the data. In response, we have revised the figure by enlarging the legend to improve legibility and ensure that all elements are clearly visible. The updated figure has been included in the revised manuscript.

**Reviewer 1 Comment 2**

2. Line 501: it was nice that the author used the phrase ‘synthesize current evidence’ as another study conducted by Moyo-Chilufya et al. 2023 considered NCD and mental disorders among PLHIV in Africa.

**Author Response to Comment 2:** Thank you for your kind words and for highlighting the relevance of the phrase in connection with the work by Moyo-Chilufya et al. (2023). We appreciate your recognition and are encouraged by the alignment of our approach with existing research in this important area.

**Reviewer 1 Comment 3**

3. Line 574: ‘As the population ages and the prevalence of NCDs rises’, kindly provide a reference for this statement.

**Author Response to Comment 3:** Thank you for catching this important detail. We have now added appropriate references to support this statement, drawing from recent literature that highlights the global trends in aging populations and the increasing burden of NCDs. These citations have been included in the revised manuscript to strengthen the contextual foundation of this point.

**Reviewer 2 Comment 1**

1. In this scoping review, the authors explored existing evidence on the relationship between HIV, mental health issues, and non-communicable co-morbidities such as hypertension and diabetes, The review is interesting and well-written. This not usually the case for me as a reviewer, but I have almost not substantive comments for the authors. The review is contextualized appropriately, the methods are described adequately, the results seem reliable and interesting, and the discussion is reasonable without being overly ambitious. Well done!

   My only suggestion would be to include a discussion of the lack of children or adolescents living with HIV in these studies. All the studies included in the review enrolled people above 18 years of age, and many were much older. Children and young people living with HIV tend to be neglected in research, especially mental health research. For a starting point for discussion of this, see the section on “insights in absence” in DOI:10.1093/braincomms/fcad231 which also found that children and young people were absent in research on depression/inflammation. I appreciate that many of the non-communicable diseases that were of interest to the authors are more prevalent in older adults – though this is not always the case (e.g. Type I diabetes and chronic respiratory issues can be of substantial impact in young people), so it’s still important to highlight the lack of evidence in children with HIV.

**Author Response to Comment 1:**  Thank you very much for your generous feedback and for highlighting an important gap in the literature. We greatly appreciate your thoughtful suggestion regarding the exclusion of children and adolescents living with HIV in the studies reviewed. In response we have expanded **Section 4.1,** to explicitly address this issue by discussing the lack of syndemic research involving younger populations living with HIV in Africa. We have also cited the article you recommended (DOI:10.1093/braincomms/fcad231) to underscore the broader concern of underrepresentation of children and adolescents in mental health and syndemic research. We agree that this is a critical area for future investigation, particularly given the unique challenges faced by younger individuals with chronic conditions such as HIV and NCDs.
