# Supplementary material for: Syndemic interactions between HIV/AIDS, mental health conditions, and non-communicable diseases in sub-Saharan Africa: A scoping review of contributing factors
Source: PLoS One. 2025 Aug 21;20(8):e0328515. doi: 10.1371/journal.pone.0328515 (PMC12370135; doi:10.1371/journal.pone.0328515)
Supplement: Attachment — Submitted filename: Response to Reviewers 2.docx [file pone.0328515.s005.docx]

**Author Responses to Editorial and Reviewer Comments.**

We would like to express our sincere gratitude to the reviewers and the editorial team for their careful and thorough review of our manuscript. We greatly appreciate the time, effort, and expertise that went into evaluating our work. In the sections below, we provide detailed, point-by-point responses to each of the editorial and reviewer comments. We have carefully considered all feedback and have revised the manuscript accordingly to address the concerns and suggestions raised.

We are especially thankful for the opportunity to revise and resubmit our manuscript. The constructive critiques we received have been instrumental in enhancing the clarity, rigor, and overall quality of the paper. We believe that the manuscript is now substantially improved and hope that the revisions meet your expectations.

**Comments from the Editor**

**Editor Comment 1**

1. **Kindly review Figure 2 (the PRISMA flow diagram) carefully to ensure that the number of studies is accurately represented throughout the chart.**

**Author Response to Comment 2:** Thank you for carefully reviewing the figure and bringing this discrepancy to our attention. We have rechecked the calculations and identified the source of the error. The figure has been revised accordingly to accurately reflect the correct number of studies. The updated version is now included in the revised manuscript.
